# Prednisolone suppresses adriamycin-induced vascular smooth muscle cell senescence and inflammatory response via the SIRT1-AMPK signaling pathway

**DOI:** 10.1371/journal.pone.0239976

**Published:** 2020-09-30

**Authors:** Jin Young Sung, Seul Gi Kim, Jae-Ryong Kim, Hyoung Chul Choi

**Affiliations:** 1 Department of Pharmacology, College of Medicine, Yeungnam University, Daegu, Republic of Korea; 2 Smart-aging Convergence Research Center, College of Medicine, Yeungnam University, Daegu, Republic of Korea; 3 Department of Biochemistry and Molecular Biology, College of Medicine, Yeungnam University, Daegu, Republic of Korea; Medical College of Georgia at Augusta University, UNITED STATES

## Abstract

Cellular senescence is associated with inflammation and the senescence-associated secretory phenotype (SASP) of secreted proteins. Vascular smooth muscle cell (VSMC) expressing the SASP contributes to chronic vascular inflammation, loss of vascular function, and the developments of age-related diseases. Although VSMC senescence is well recognized, the mechanism of VSMC senescence and inflammation has not been established. In this study, we aimed to determine whether prednisolone (PD) attenuates adriamycin (ADR)-induced VSMC senescence and inflammation through the SIRT1-AMPK signaling pathway. We found that PD inhibited ADR-induced VSMC senescence and inflammation response by decreasing p-NF-κB expression through the SIRT1-AMPK signaling pathway. In addition, Western blotting revealed PD not only increased SIRT1 expression but also increased the phosphorylation of AMPK at Ser485 in ADR-treated VSMC. Furthermore, siRNA-mediated downregulation or pharmacological inhibitions of SIRT1 or AMPK significantly augmented ADR-induced inflammatory response and senescence in VSMC despite PD treatment. In contrast, the overexpression of SIRT1 or constitutively active AMPKα (CA-AMPKα) attenuated cellular senescence and p-NF-κB expression. Taken together, the inhibition of p-NF-κB by PD through the SIRT1 and p-AMPK (Ser485) pathway suppressed VSMC senescence and inflammation. Collectively, our results suggest that anti-aging effects of PD are caused by reduced VSMC senescence and inflammation due to reciprocal regulation of the SIRT1/p-AMPK (Ser485) signaling pathway.

## Introduction

Vascular aging causes various pathological alterations in blood vessels, including chronic inflammation, vascular cell phenotypic shifts, and structural modifications, which lead to aging-associated diseases such as cardiovascular disease and atherosclerosis [1]. Cellular senescence is also characterized by changes in morphology and function. The features of cellular senescence are mainly altered cell morphology, increased activity of senescence associated-β-galactosidase (SA-β-gal), accumulation of DNA damage, and increased expression level of p21 and p53 [2]. Adriamycin (ADR) has been used as a common chemotherapeutic drug for decades [3]. In HT1080 cells, the strongest induction of the senescent phenotype was seen with ADR [4]. In addition, ADR induced senescence in tumor cells, including p53 and p21 overexpressed cells [5]. Therefore, ADR induce senescent characteristics in cells and is more efficient in generating senescence in cell culture [6]. We suggest that ADR-induced senescence can be observed in vascular smooth muscle cell (VSMC). The senescent cells also develop the senescence associated secretory phenotype (SASP), which induces inflammation and senescence [7, 8]. Senescence-associated inflammation is caused by the secretions of pro-inflammatory molecules and is associated with many age‐related diseases like atherosclerosis [9, 10]. VSMC senescence in vasculature is induced by inflammatory reactions regulated by nuclear factor-κB (NF-κB) [11], and it has been reported inhibition of inflammatory reactions in VSMC senescence can prevent the developments of aging-associated diseases and reverse the natural features of aging [12]. Therefore, an understanding how senescence drives inflammation is of critical importance.

Glucocorticoid (GC) is well-known anti-inflammatory agent that is widely used to treat inflammatory diseases [13]. GC has potent anti-inflammatory activities and suppresses inflammation mainly by activating or repressing genes encoding anti-inflammatory or pro-inflammatory cytokines, respectively [14]. Prednisolone (PD) is a synthetic corticosteroid with predominant glucocorticoid activity and is used for the treatment of inflammatory conditions including asthma [15]. However, despite its excellent anti-inflammatory efficacies, the mechanism responsible for their effects in the context of inflammation-induced cellular senescence in vascular disease has not been determined.

NF-κB plays a key role in the expression of many inflammatory transcription factor associated genes [16]. In arteries, NF-κB is believed to promote cardiovascular diseases by enhancing the transcriptions of pro-inflammatory and pro-oxidant genes [17]. Furthermore, previous studies have shown NF-κB activity is higher in the heart, liver, kidney and brain tissues of old rodents than in young rodents [18, 19]. Moreover, treatment with GC attenuates NF-κB expression and confers significant vasoprotective effects in age-related cardiovascular diseases in animal models and in man. GC also has been reported to inhibit the NF-κB cellular pathway [20, 21]. However, its effects on the inflammation of senescent VSMC have not been characterized.

Crosstalk between sirtuin 1 (SIRT1) and cAMP-activated protein kinase (AMPK) plays a central role senescence [22, 23]. SIRT1 (a NAD-dependent histone deacetylase) is a member of the highly conserved sirtuin family that regulates multiple biological processes such as cellular metabolism, aging, and stress response [24, 25]. Other studies have demonstrated SIRT1 has an inhibitory effect on NF-κB-mediated inflammation, and that resveratrol-induced SIRT1 overexpression or activation promotes the deacetylation of p65 and suppresses transcriptional activation by NF-κB [26]. On the other hand, gene knockdown or inhibition of SIRT1 potentiates inflammatory processes via the SIRT1-mediated deacetylation of p65 [27]. AMPK is also associated with cellular senescence as evidenced by AMP/ATP ratio and AMPK activity elevations in multiple tissues [28]. AMPK is generally activated when the AMP/ATP ratio is high with p-AMPK (Thr172) [29]. Although p-AMPK (Thr172) is regarded as the main phosphorylation site on AMPK, changes in AMPK activity are often observed in the absence of altered Thr172 phosphorylation [30]. The phosphorylation of AMPK at Ser485 by PKA alters the accessibility of p-AMPK (Thr172) accessibility, but roles of AMPK phosphorylation sites in AMPK activity have not been fully clarified [31, 32] or comprehensively identified in the context of VSMC senescence and inflammation.

In this study, we investigated whether VSMC senescence might be alleviated by inhibiting inflammatory response via SIRT1 and AMPK signaling pathway using PD *in vitro* and *in vivo*. We hypothesized that PD could stimulate SIRT1 and AMPK activities in VSMC, and thus, attenuate ADR-induced inflammatory response.

## Materials and methods

### Reagents and antibodies

Prednisolone (PD), doxorubicin (adriamycin, ADR) and EX527 (a selective SIRT1 inhibitor) were supplied by Sigma (St. Louis, MO, USA). Compound C (a specific AMPK inhibitor) was obtained from Calbiochem (La Jolla, CA, USA). Antibodies were purchased from the following vendors: SIRT1 (#8469), p-AMPK (Ser485, #4184), AMPK (#2532), p-NF-κB (Ser536, #3033), NF-κB (#3034), IκB (#9242) and p53 (# 2524) (Cell Signaling Technology, Danvers, MA, USA), SMP30 (sc-390098) and β-actin (sc-58673) (Santa Cruz, Delaware CA, USA).

### Cell culture and Western blot analysis

We euthanized 8-week-old male Sprague-Dawley (SD) rat with 95% CO_2_, and VSMC was isolated from the thoracic aorta. The cell was processed using a 1-mm chop setting in a 10 cm culture dish, and cultured with 50% FBS-Dulbecco's modified Eagle's medium (DMEM) with 1% antibiotics in a CO2 incubator (5% CO_2_/95% air, 37°C). SD rat aortic VSMC from passage 4 to 8 was seeded at equal densities (30 μg/μl) and cultured in high glucose DMEM (HyClone, Logan, UT, USA) supplemented with 10% FBS (HyClone, Logan, UT, USA) and 50 U/ml penicillin. Cell was then lysed in RIPA lysis buffer supplemented with 0.01 mM PIC, incubated on ice for 10 min, and centrifugated at 13,000 g for 20 min at 4°C. Protein concentration in centrifuged supernatant was determined by Bradford assay (Bio-Rad Lab, Hercules, CA, USA). For Western blotting, protein was separated by sodium dodecyl sulfate- polyacrylamide gel electrophoresis and transferred to polyvinylidene difluoride membranes, which were then immunoblotted with indicated primary antibodies followed by corresponding secondary antibodies (1:5000). Signals were visualized using chemiluminescence detection regents (Millipore, Billerica, MA, USA), according to the manufacturer’s instructions.

### Immunohistochemical staining

VSMC was seeded on 6-well plates, washed, pre-incubated with 0.3% hydrogen peroxide in methanol, washed with PBS-T, blocked with blocking buffer for 1 hr, and incubated with primary antibodies against SIRT1 and p-NF-κB (Ser536) at a dilution of 1:50 overnight at 4°C. Cell was then washed with PBS-T, incubated with HRP-labeled secondary antibody (1:1000) for 1 hr at room temperature (RT), and rewashed. Bound complexes were visualized by apply DAB Kit solution (Thermo Scientific, Waltham, MA, USA).

### Immunofluorescence analysis

VSMC was fixed with 4% buffered paraformaldehyde for 30 min, permeabilized with 0.2% Triton X-100 for 5 min at RT, blocked with 5% normal goat serum in PBS-0.05% Tween 20 (PBS-T), and incubated with anti-SIRT1, p-AMPK (Ser485) and p-NF-κB (Ser536) primary antibodies (1:100) overnight at 4°C. Cell was then treated with Alexa Fluor® 488 goat anti-rabbit and 546 goat anti-mouse secondary antibodies at 1:200 (Invitrogen, Carlsbad, CA, USA) for 45 min at RT. Nuclei were then stained with DAPI for 10 min at RT, and cells observed under an Olympus microscope equipped with LMPLFLNM Plan FL 20x lenses (Olympus, Tokyo, Japan).

### Senescence-associated β-galactosidase (SA-β-gal) staining

Senescence-associated β-galactosidase (SA-β-gal) staining was performed as previously described [33]. VSMC was seeded on 6-well plates, fixed with 4% formaldehyde for 30 min at RT, washed with PBS, and stained using a senescence-associated β-gal staining Kit (Cell Signaling Technology, Danvers, MA, USA). Percentages of blue cells per 100 cells observed under a light microscope at ×100 were determined.

### SIRT1 activity assay

The SIRT1 activity assay was performed using a commercial fluorogenic SIRT1 Assay Kit (BPS Bioscience, San Diego, CA, USA). Final reaction mixture (50 μl) contained 100 μM SIRT1 substrate, 1 mg/ml BSA, 50 mM NAD+, HDAC assay buffer, 25 ng/μl SIRT1, and 5 μl of lysed test sample and incubated for 30 min at 37°C. The reactions were stopped by adding a developer, which generated a fluorophore. Fluorescence intensities were measured at excitation and emission wavelengths of 350 and 460 nm, respectively, using a fluorometric plate reader (Bio-Rad Lab, Hercules, CA, USA).

### Immunoprecipitation analysis

Cell lysates were incubated with anti-SIRT1 or anti-rabbit IgG (Santa Cruz, Delaware, CA, USA) antibody overnight at 4°C, as described previously [34]. Protein A/G beads were added to the immunocomplex and inbubated for 4 hr at 4°C. Unbound proteins were removed by centrifugation, and co-immunoprecipitation was determined by using Western blot analysis.

### Transfection of siRNA and plasmid DNA

VSMC was transfected with control siRNA or siRNA against SIRT1 and AMPK by using Lipofectamine 2000 Reagent (Invitrogen, Carlsbad, CA, USA), according to the manufacturer’s instructions. Con siRNA, SIRT1 siRNA, and AMPK siRNA were purchased from Santa Cruz (USA). Cells then were resuspended in complete DMEM, incubated for 24–48 hr, and used for further experiments. Flag-SIRT1 was obtained from Addgene plasmid repository (Addgene plasmid #1791; Greenberg ME, Harvard Medical School, Boston, MA, USA). For transfection, VSMC was seeded on 6-well plates and when 80% confluent, transfected in Opti-MEM/Lipofectamine 2000 Reagent containing SIRT1 plasmid DNA.

### Adenoviral vector and transduction

Adenoviruses expressing GFP (the control gene) and a constitutively active form of AMPKα (CA-AMPKα) were obtained from Dr. In-Kyu Lee (Kyungpook National University School of Medicine) and amplified in VSMC using standard methodologies. VSMC was transfected in serum-free DMEM over 6 hr.

### Mice experimental design

C57BL/6 male mice were purchased from Koatech (Gyeonggi-do, Republic of Korea). Mice were maintained under specific pathogen–free conditions at the laboratory animal care unit, Yeungnam University College of Medicine and condition of the animals was monitored every 3 days. Male mice (25–30 g body weight) were divide into four groups of three mice: (1) a saline treated group (control group), (2) an ADR (a single 10 mg/kg i.p. injection in deionized water) group (ADR group), (3) an ADR plus PD (three 10 mg/kg i.p. injection in DMSO) group (ADR plus 3PD group), and (4) an ADR plus PD (a single 10 mg/kg i.p. injection) group (ADR plus 1PD group). Mice were administered ADR on experimental day 1 (ED1), and mice were administered a single injection of PD once every 2 days and on ED6. All mice were sacrificed on ED7. We euthanized C57BL/6 male mice with 95% CO_2_, and thoracic aorta was isolated. Animal experiments were approved by the Institutional Animal Care and Use Committee at Yeungnam University College of Medicine.

### Statistical analysis

Results in the bar graph are the means ± SEM for three independent experiments. The significance of differences between two groups was determined using the Student’s t test, and multiple group comparisons were performed by ANOVA followed by Bonferroni’s *post hoc* test. The analysis was conducted using GraphPad Prism8 (Graph-Pad Software Inc.), and statistical significance was accepted for *p* values of < 0.05.

## Results

### Prednisolone delays VSMC senescence by inhibiting p-NF-κB

**Fig 1:**
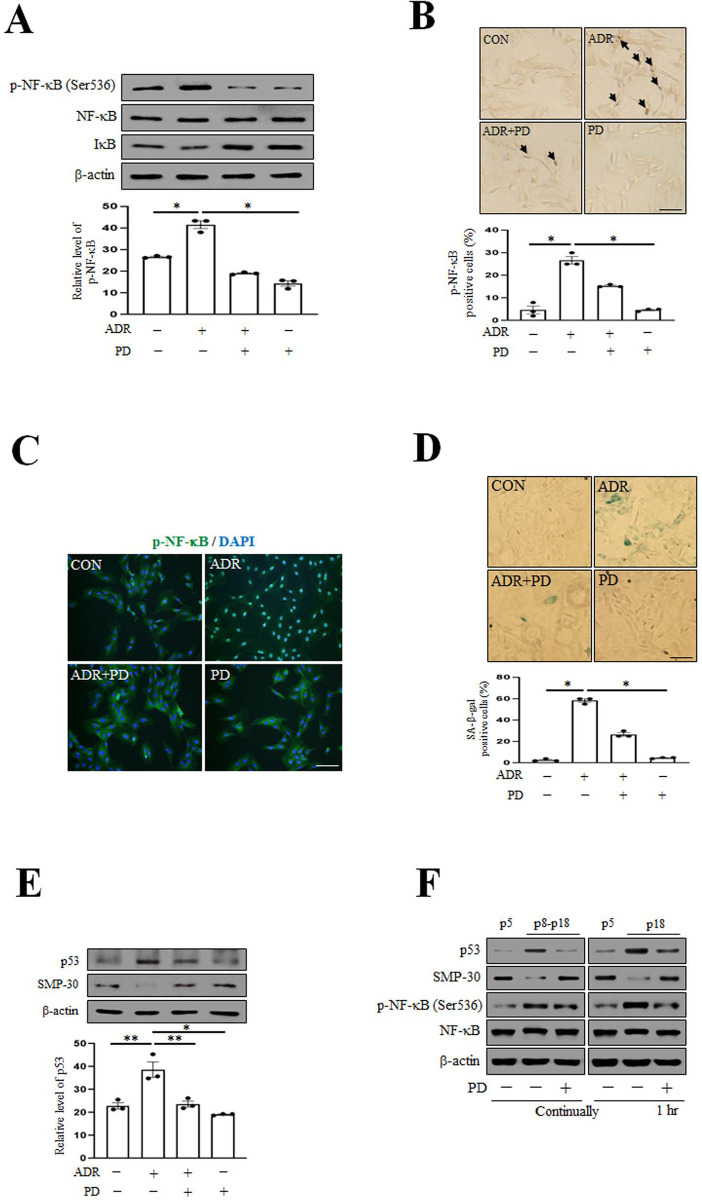
Prednisolone delays VSMC senescence caused by vascular inflammation. (A) VSMC was exposed to adriamycin (ADR, 500 nM) for 4 hr and then treated with prednisolone (PD, 10 μM) for 1 hr. Protein expression levels were analyzed by immunoblotting. (B) VSMC was exposed to ADR and then treated with PD. After incubation, VSMC was stained for DAB (brown) using antibodies against p-NF-κB (1:50). Images were taken at an original magnification of ×100. Scale bar, 100 μm. Percentages of brown cells per 100 cells were determined. (C) VSMC phenotypes were determined by indirect immunofluorescent staining with p-NF-κB antibodies (1:100). Nuclei were stained with DAPI. The images were taken at an original magnification of ×200. Scale bar, 100 μm. (D) After treatment with ADR and PD, cellular senescence was examined by SA-β-gal staining (senescent cells were stained blue) and examined by bright-field microscopy (original magnification ×100). Scale bar, 100 μm. Percentages of blue cells per 100 cells were was determined. (E) VSMC was exposed to ADR and then treated with PD and protein expression levels were analyzed by immunoblotting using antibodies against p53, SMP-30, β-actin. (F) VSMC was treated with PD for continually during passage (p) 8 to p18 or at p18 for 1 hr, and p53, SMP30, p-NF-κB, NF-κB, and β-actin levels were assessed by Western blotting. Results are expressed as the means±SEM of three independent experiments. Statistical analysis was performed with Student’s t test, and ANOVA followed by Bonferroni’s post hoc test. *p<0.01, **p<0.05.

NF-κB activation has been associated with age-related disease [35, 36]. Thus, we investigated whether prednisolone (PD) could alleviate VSMC senescence by inhibiting NF-κB phosphorylation by examining p-NF-κB levels in ADR-treated VSMC. We found PD decreased ADR-induced inflammation (Fig 1A and 1B), and suppressed the nuclear translocation of p-NF-κB from cytosol by immunofluorescent analysis (Fig 1C). In addition, we investigated whether PD suppresses ADR-induced VSMC senescence by staining with SA-β-gal [33], a marker of senescent cells. As shown in Fig 1D and 1E, PD reduced the number of SA-β-gal positive cells and p53 levels, but increased levels of SMP-30 (an anti-aging protein) [37]. Furthermore, replicative cellular senescence in cell culture is dependent on the number of cell divisions, as occurs during natural aging [38], we investigated the anti-aging effect of PD on natural aging. PD (10 μM) was administered for continually during p8- p18 or at p18 for 1 hr, and then levels of p53, SMP30, p-NF-κB and NF-κB were assessed by Western blotting. The results of continually and short-term PD treatment were similar (Fig 1F). Taken together, these results indicate that PD suppressed expressions of p-NF-kB, p53 and reduced SA-β-gal positive cells.

### Prednisolone reduces VSMC senescence through the SIRT1-AMPK signaling pathway

**Fig 2:**
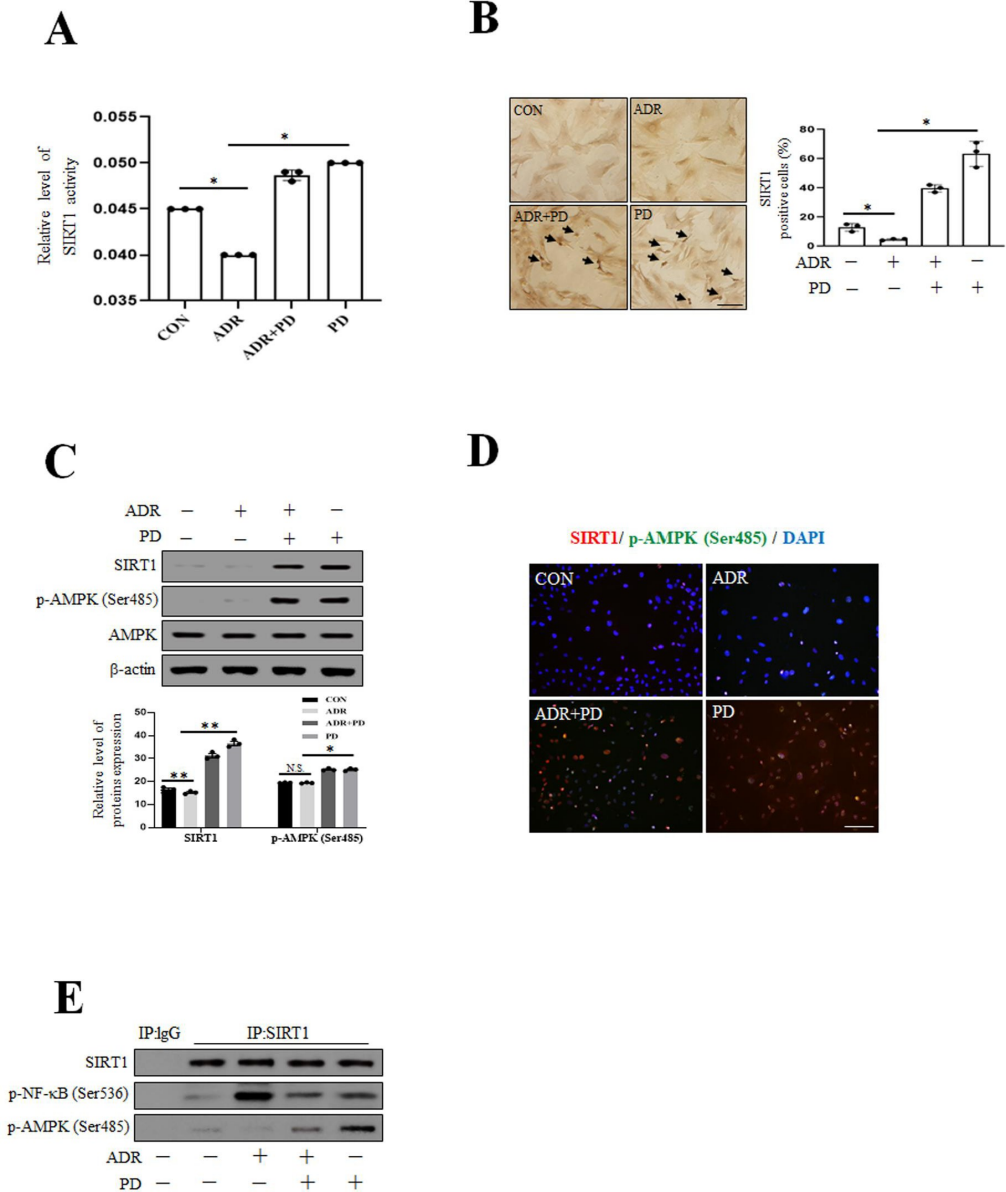
Prednisolone increases the activation of SIRT1 and AMPK and reduced inflammation-induced VSMC senescence. (A) VSMC was exposed to ADR for 4 hr and then PD for 1 hr, and intracellular SIRT1 activity was measured using a fluorogenic SIRT1 assay kit. (B) After treatment with ADR and PD, VSMC was stained with DAB (brown) using SIRT1 antibody (1:50). Images were taken at an original magnification of ×100. Scale bar, 100 μm. Percentage of brown cells per 100 cells observed under a light microscope at ×100 were calculated. (C) VSMC was exposed to ADR and then treated with PD, and protein expression levels were analyzed using immunoblotting. (D) After treatment with ADR and PD, VSMC phenotypes were determined by indirect immunofluorescent staining with antibodies against SIRT1 and p-AMPK (Ser485) (1:100). Nuclei were stained with DAPI. Images were taken at an original magnification of ×200. Scale bar, 100 μm. (E) VSMC was subjected to immunoprecipitation analysis using SIRT1 antibody or control IgG, and then blotted with antibodies-recognizing p-NF-κB and p-AMPK (Ser485). Total SIRT1 levels in whole cell lysates were estimated by immunoblotting. Results are expressed as the means±SEM of three independent experiments. Statistical analysis was performed with Student’s t test, and ANOVA followed by Bonferroni’s post hoc test. *p<0.01, **p<0.05, N.S. (Not significant).

Previous studies have suggested that the activation of SIRT1 and AMPK has anti-aging effect [39, 40]. We first examined the effects of ADR and ADR plus PD on deacetylase activity and expression level of SIRT1. As shown in Fig 2A and 2B, ADR-induced reduction of the activity and expression level of SIRT1 was reversed by PD. In addition, PD increased the expression levels of SIRT1 and p-AMPK (Ser485) as compared with treating ADR alone (Fig 2C and 2D). We also examined interactions between SIRT1 and NF-κB by immunoprecipitation analysis (Fig 2E) and found PD blocked the expression of p-NF-κB, which is known to promote cellular senescence, but increased the expression of p-AMPK (Ser485). Collectively, these observations show PD induced SIRT1 activity and p-AMPK (Ser485) expression but suppressed p-NF-κB expression.

### Inhibition of SIRT1 and AMPK attenuates the anti-aging effect of prednisolone on ADR-induced VSMC senescence

**Fig 3:**
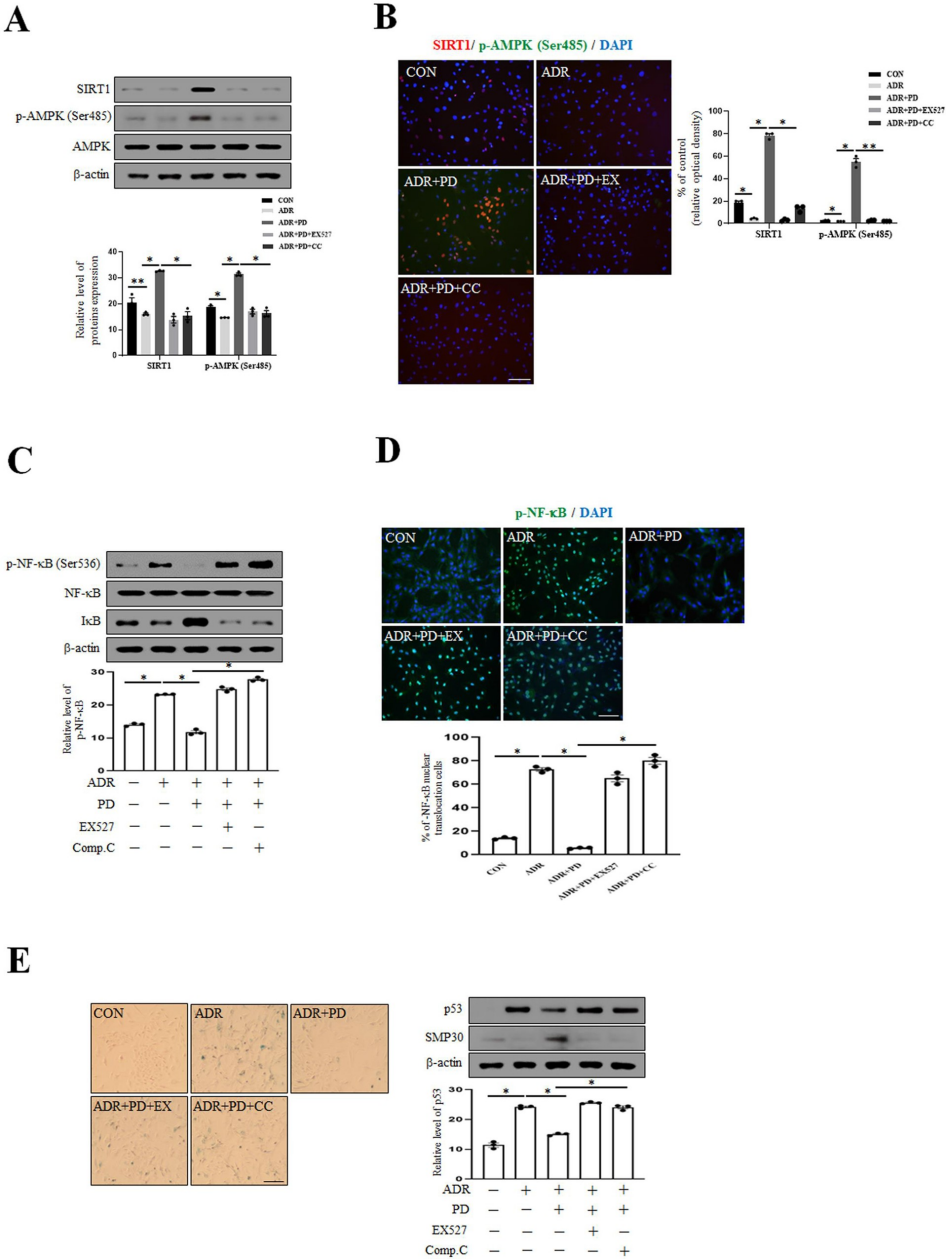
Inhibitors of SIRT1 and AMPK reverses the senescent VSMC phenotype. (A) VSMC was exposed with ADR for 4hr and after then pretreated EX527 (10 μM) or compound c (Comp.C, 10 μM) for 1 hr, and treated with PD (10 μM) for 1 hr. Protein expression levels were analyzed by immunoblotting. (B) After treatment with all drug, respectively, VSMC phenotypes were determined by indirect immunofluorescent staining with antibodies for SIRT1 and p-AMPK (Ser485) (1:100). Nuclei were stained with DAPI. The images were taken at an original magnification of ×200. Scale bar, 100 μm. (C) VSMC was exposed with ADR for 4hr and after then pretreated EX527 (10 μM) or compound c (Comp.C, 10 μM) for 1 hr, and treated with PD (10 μM) for 1 hr. Protein expression levels were assessed by immunoblotting. (D) VSMC phenotypes were identified by indirect immunofluorescent staining with antibodies for p-NF-κB (1:100). Nuclei were stained with DAPI. Images were taken at an original magnification of ×200. Scale bar, 100 μm. (E) After treatment with all drug, cellular senescence was examined by SA-β-gal staining and p53 and SMP-30 levels were estimated by Western blotting. Images were taken at an original magnification of ×100. Scale bar, 100 μm. Results are expressed as the means±SEM of three independent experiments. Statistical analysis was performed with Student’s t test, and ANOVA followed by Bonferroni’s post hoc test. *p<0.01, **p<0.05.

Given that the activity of SIRT1 and the expression level of p-AMPK (Ser485) were increased by PD, we tested whether their inhibitions reduced anti-aging effect of PD on ADR-induced VSMC senescence. The expression levels of SIRT1 and p-AMPK (Ser485) were reduced by EX527 and compound c despite PD treatment (Fig 3A and 3B). However, the expression level of p-NF-κB was increased by treating SIRT1 and AMPK inhibitors regardless of PD treatment in VSMC (Fig 3C and 3D). We also investigated whether their inhibitors affect VSMC senescence. As shown in Fig 3E, the inhibition of SIRT1 and AMPK significantly increased the expression level of p53 and SA-β-gal positive cell numbers in the presence of absence of PD. These findings indicate that SIRT1 and AMPK inhibit p-NF-κB and attenuate anti-aging effect of PD on ADR-induced VSMC senescence.

### Knockdown of SIRT1 and AMPK abolishes the anti-aging effect of prednisolone by increasing p-NF-κB

**Fig 4:**
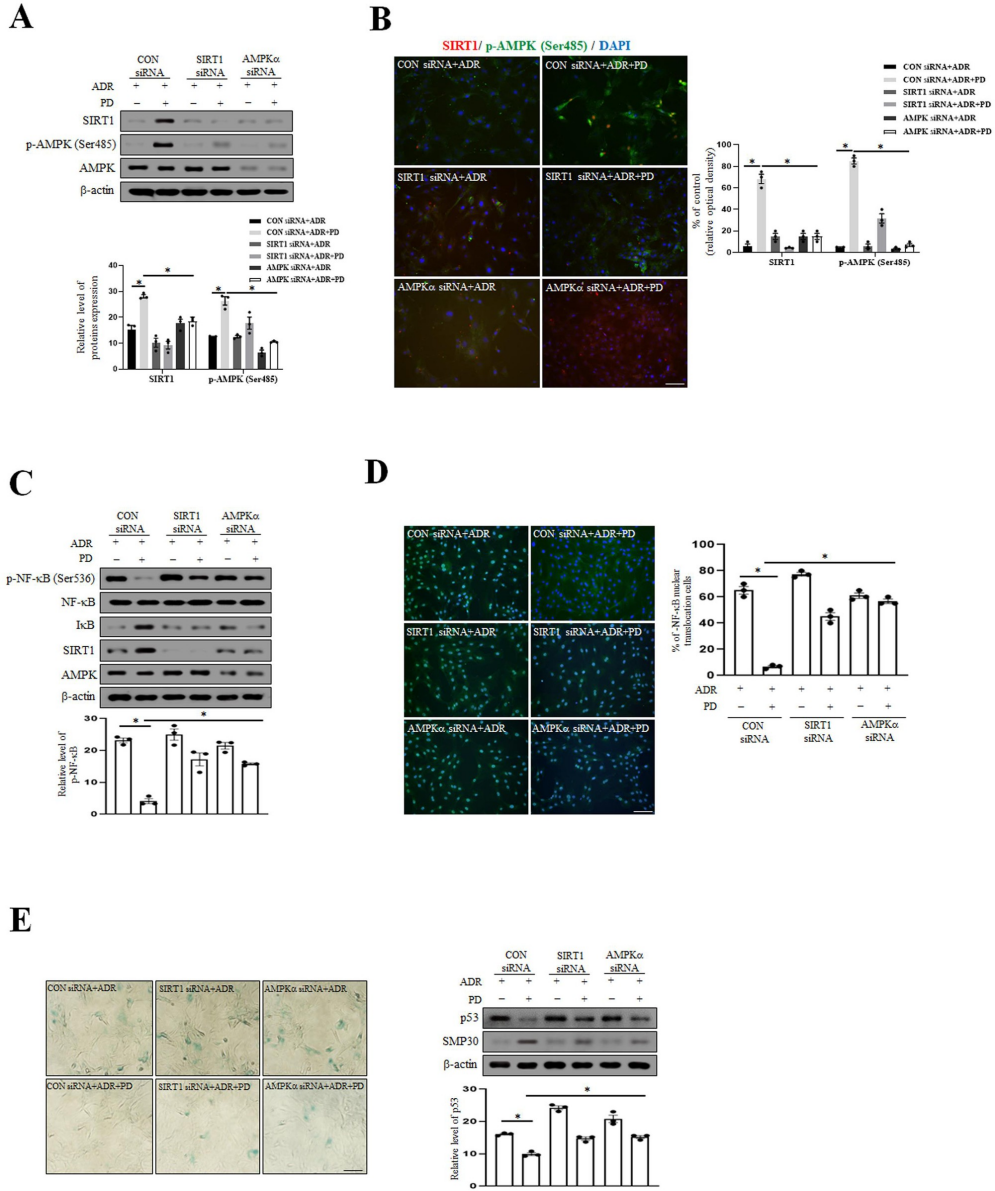
Knockdown of SIRT1 and AMPK increases VSMC senescence by increasing p-NF-κB. (A) VSMC was transfected with control siRNA, SIRT1 siRNA and AMPKα siRNA (10 μM) for 48 hr, exposed to ADR (500 nM) for 4 hr, and then treated with PD (10 μM) for 1hr. Protein expression levels were assessed by immunoblotting. (B) After transfection and treatment with ADR and PD, VSMC phenotypes were identified by indirect immunofluorescent staining with antibodies for SIRT1 and p-AMPK (Ser485) (1:100). Nuclei were stained with DAPI. Images were taken at an original magnification of ×200. Scale bar, 100 μm. (C) After transfection of siRNAs and treatment of ADR and PD, protein expression levels were analyzed by immunoblotting. (D) VSMC phenotypes were identified by indirect immunofluorescent staining with antibodies for p-NF-κB (1:100). Nuclei were stained with DAPI. Images were taken at an original magnification of ×200. Scale bar, 100 μm. (E) After all drug treatment, cellular senescence was examined by SA-β-gal staining and p53 and SMP-30 levels were analyzed by Western blotting. Images were taken at an original magnification of ×100. Scale bar, 100 μm. Results are expressed as means±SEM of three independent experiments. Statistical analysis was performed with Student’s t test, and ANOVA followed by Bonferroni’s post hoc test. * *p*<0.01.

After knocking out the SIRT1 and AMPK gene, we investigated the molecular mechanisms underlying the anti-aging effect of PD. As shown in Fig 4A and 4B, the transfections of SIRT1 and AMPKα siRNAs were successful. Furthermore, these knockdowns increased the expression level of p-NF-κB versus control siRNA transfected cells despite PD treatment (Fig 4C and 4D). Moreover, knockdown of SIRT1 and AMPKα increased SA-β-gal positive cell numbers and the expression levels of p-NF-κB and p53 in ADR-induced VSMC senescence (Fig 4E). These results suggest the SIRT1 and p-AMPK (Ser485) pathway plays an important role in VSMC senescence by decreasing p-NF-κB levels.

### The overexpression of SIRT1 and AMPK attenuates VSMC senescence by inhibiting p-NF-κB

**Fig 5:**
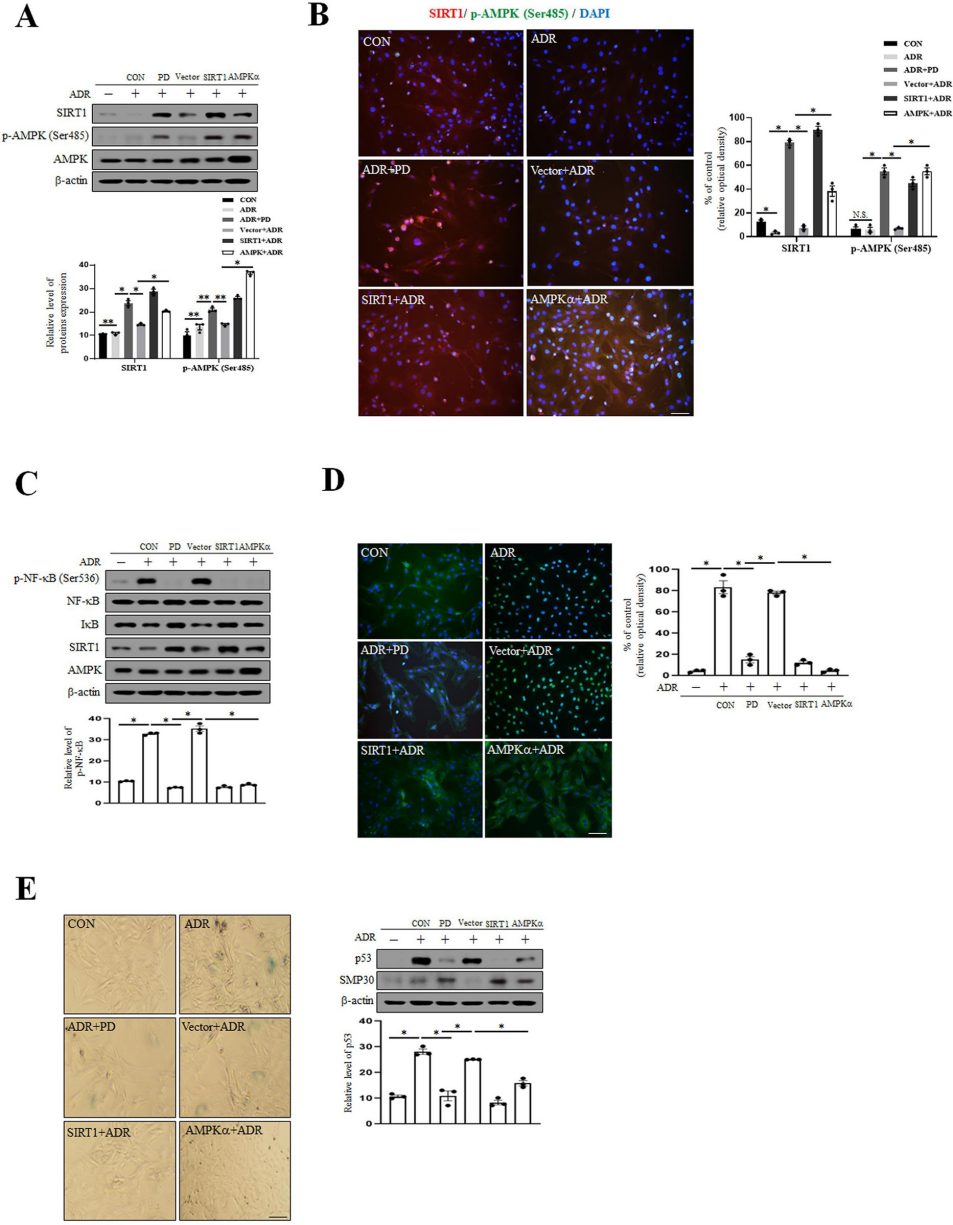
ADR-induced NF-κB activation is abolished by the overexpression of SIRT1 and AMPK in senescent VSMC. (A) VSMC was transduced with Xp-His vector or flag-SIRT1 plasmid and CA-AMPKα to overexpress SIRT1 and AMPK. After infection for 24 hr, VSMC was exposed to ADR (500 nM) for 4 hr and then treated with PD (10 μM) for 1 hr. Protein expression levels were assessed by immunoblotting. (B) After transduction and treatment of ADR and PD, VSMC phenotypes were determined by indirect immunofluorescent staining with antibodies for SIRT1 and p-AMPK (Ser485) (1:100). Nuclei were stained with DAPI. Images were taken at an original magnification of ×200. Scale bar, 100 μm. (C) After transduction and treatment of ADR and PD, protein expression levels were assessed by immunoblotting. (D) VSMC phenotypes were determined by indirect immunofluorescent staining with antibodies for p-NF-κB (1:100). Nuclei were stained with DAPI. Images were taken at an original magnification of ×200. Scale bar, 100 μm. (E) Cellular senescence was examined by SA-β-gal staining and the p53 and SMP-30 levels were estimated by Western blotting. Images were taken at an original magnification of ×100. Scale bar, 100 μm. Results are expressed as the means±SEM of three independent experiments. Statistical analysis was performed with Student’s t test, and ANOVA followed by Bonferroni’s post hoc test. *p<0.01, **p<0.05, N.S. (Not significant).

To confirm SIRT1 and AMPK activities are required to inhibit ADR-induced VSMC senescence and the expression level of p-NF-κB, we examined whether the effects of overexpressing the SIRT1 and AMPKα gene. As shown in Fig 5A and 5B, SIRT1 overexpression by SIRT1 plasmid and AMPKα overexpression (CA-AMPKα) markedly increased the expression levels of SIRT1 and p-AMPK (Ser485), respectively. In addition, we examined the expression level of p-NF-κB by Western blot and immunofluorescent analysis. The p-NF-κB in ADR treated VSMC was reduced by overexpression of SIRT1 and AMPK (Fig 5C and 5D). In ADR treated VSMC, treatment with Ad-SIRT1 and CA-AMPKα remarkably decreased SA-β-gal positive cell counts and reduced the expression level of p53 (Fig 5E). These results show that the activations of SIRT1 and AMPK alleviate VSMC senescence by inhibiting p-NF-κB.

### Prednisolone enhances the expression of SIRT1/p-AMPK (Ser485) and reduces the expression of p-NF-κB/p53 in aged model

**Fig 6:**
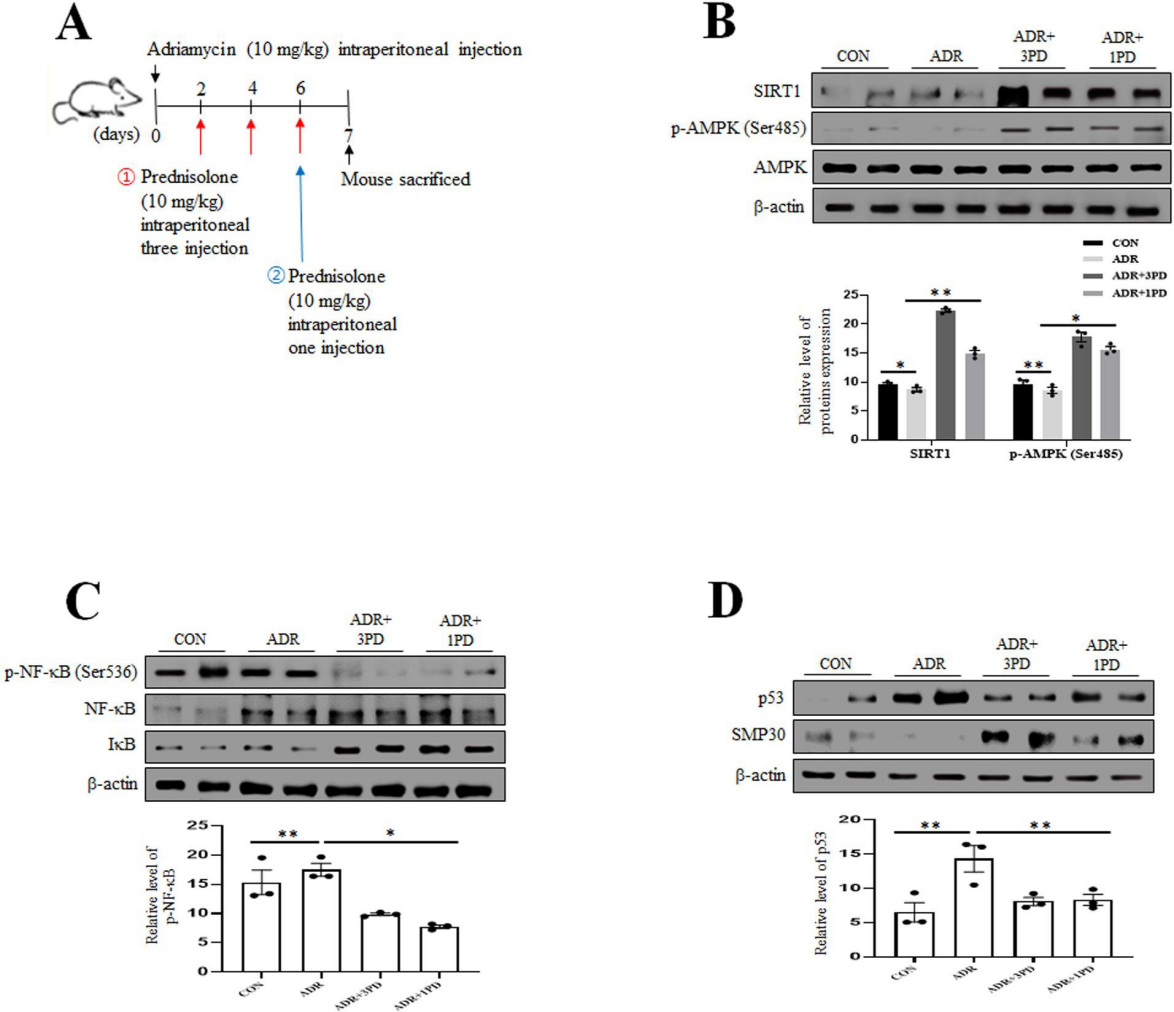
Prednisolone inhibits p-NF-κB and increase SIRT1 and AMPK expression levels in ADR treated mice. (A) Mice were divided into four groups of three mice: (1) the control group (CON), (2) the ADR group (ADR), (3) the ADR plus three PD (ADR+3PD), and (4) ADR plus a single PD (ADR+1PD). ADR (10 mg/kg of body weight) and PD (10 mg/kg of body weight) were injected intraperitoneally. Mice in the ADR group were injected with ADR on experimental day 1 (ED1), and mice in the ADR+3PD group were injected with PD once every 2 days or mice in the ADR+1PD group were injected a single on ED6. All mice were sacrificed on ED7 after ADR administration. (B, C, and D) Immediately after sacrifice, aortas were extracted from mice in the four group and protein expression levels were analyzed using immunoblotting. (C) After the aorta extracted, protein levels in aortic tissues were assessed by immunoblotting. (D) After the aorta extracted protein expression levels were analyzed using immunoblotting. Results are expressed as the means±SEM of three independent experiments. Statistical analysis was performed with Student’s t test, and ANOVA followed by Bonferroni’s post hoc test. *p<0.01, **p<0.05, N.S. (Not significant).

The aortas of mice treated with ADR and ADR plus PD were examined by Western blot. As shown in Fig 6B, the expression levels of SIRT1 and p-AMPK (Ser485) were markedly higher in the ADR plus PD groups than in the ADR group. Furthermore, the expression level of p-NF-κB was higher in the ADR group than in the ADR plus PD groups (Fig 6C). In the senescence state, the expression level of p53 was higher in the ADR group, but the expression level of SMP-30 was decreased (Fig 6D). Overall, these results show that PD activated SIRT1/p-AMPK (Ser485) and reduced expression of p-NF-κB, and suggest these activations play an important role in VSMC senescence and inflammation of ADR treated aorta.

## Discussion

In the current study, we investigated whether inhibiting inflammatory reactions might alleviate vascular smooth muscle cell (VSMC) senescence. Our data demonstrate that prednisolone (PD) ameliorated VSMC senescence caused by adriamycin (ADR), and show PD requires the SIRT1/p-AMPK (Ser485) signaling pathway to alleviate VSMC senescence caused by inflammation.

Aging has a major effect on the heart and arterial system, leading to an increase in cardiovascular disease (CVD) such as atherosclerosis and hypertension [41]. The accumulation of senescent cells is a hallmark of aging, one of which is inflammation. In addition, inflammation plays an important role in senescent VSMC [42, 43]. Therefore, inflammation is a promising strategy not only to prevent CVD but also to slow the decline of health that occurs with aging. In this present study, VSMC was exposed to the ADR-induced inflammation [44] as well as senescence [45]. We examined whether inhibition of ADR-induced inflammation might alleviate VSMC senescence. PD reduced the expression level of p-NF-κB in ADR-treated VSMC (Fig 1). In addition, VSMC cultured to p8-p18 and p18 showed the same results with continually and short-term treatment of PD in natural aging, which suggests PD alleviates VSMC senescence caused by inflammatory reactions, regardless of the duration of PD treatment. Taken together, these results indicate that PD can ameliorate VSMC senescence by inhibiting p-NF-κB.

In clinical practice, glucocorticoid (GC) is an important therapy due to its anti-inflammatory, anti-proliferative, and immunosuppressive properties and has been used to ameliorate inflammatory conditions associated with organ transplant rejection and respiratory and gastrointestinal diseases [46]. Although PD has previously been associated with immunosuppressive effects in endothelial cells [47], the mechanism responsible for its inhibition of the inflammatory phenotype in senescent cells has not been investigated. Thus, we investigated whether PD inhibits NF-κB activation in senescent VSMC and whether this inhibition is associated with the upregulation of SIRT1/p-AMPK (Ser485) signaling.

In this study, PD markedly increased the activity (Fig 2A) and protein expression (Fig 2C) of SIRT1. PD has been shown to activate SIRT1 and increase the expression of SIRT1 and then suppress inflammation via inhibiting the phosphorylation of NF-κB. In previous study, GC elicit their functions by binding to intracellular receptor (GR), GR dependent transactivation is crucial in the anti-inflammatory activities of glucocorticoid [48]. GC GR complex can regulate the expression of target genes, either by binding to GC response elements or inhibiting to other DNA-bound transcription factors [49]. Thus, the activity and expression of SIRT1 appears to be affected by PD (GC agonist).

SIRT1 plays a critical protective role against aortic stiffness by reducing DNA damage and cellular senescence in VSMC [50, 51], and has been shown to inhibit NF-κB-mediated inflammatory factor in in renal proximal tubule cells [52]. The NAD^+^-dependent protein deacetylase SIRT1 has been shown to regulate metabolism and inflammatory response in age-related diseases through deacetylation of key transcription factor and protein such as NF-κB [53]. Liu et al. reported that SIRT1 activation by resveratrol inhibited NF-κB activation [54] and that SIRT1 activators suppress inflammatory responses by inhibiting NF-κB activity and increasing of p65 deacetylation [55]. SIRT1 can deacetylate p65 at lysine 310 and regulate NF-kB, and SIRT1 inhibition can decrease NF-κB-mediated inflammatory response (pro-inflammatory cytokines) via SIRT1-mediated deacetylation of p65 [26, 56]. Therefore, SIRT1 is considered an important therapeutic target in inflammatory diseases. On the basis of previous studies about SIRT1-NF-κB, we identified that PD alleviated ADR-induced senescence and inflammation by decreasing p-NF-κB expression through SIRT1/p-AMPK (Ser485) signaling pathway (Figs 3–5). AMPK, which is considered to have anti-aging effects, may also play essential roles in the aging process by participating in an integrated signaling network [57].

In previous studies, Xiang et al. showed AMPK activation by AICAR (an AMPK activator) attenuates complete Freund’s adjuvant (CFA)-induced inflammatory pain by inhibiting NF-κB activity in mouse model [58]. Metformin (an AMPK activator) has been observed to suppress cytokine-induced NF-κB activation, and this suppression was markedly attenuated by transfection of AMPK siRNA into endothelial cells [59]. Moreover, it is known that AMPK signaling pathway interacts SIRT1 to suppress NF-kB signaling [60]. In addition, we previously reported that SRT1720-induced activation of SIRT1 alleviates VSMC senescence via cAMP/PKA-dependent p-AMPK (Ser485) [45]. In other study, it also was reported that adiponectin inhibits inflammatory response through cAMP-PKA/AMPK/NF-κB axis and inflammatory stimuli could reduce cAMP levels, PKA activity, and p-AMPK levels in vascular diseases [61]. Therefore, the aim of the present study was to examine whether PD regulates the expression of p-NF-kB via SIRT1/p-AMPK (Ser485) signaling pathway. Our observation of a novel connection between ADR-induced VSMC senescence and inflammation suggests the inhibition of p-NF-κB level and activity of PD-induced SIRT1/p-AMPK (Ser485) signaling pathway. Current result show that decreased AMPK protein expression (p-AMPKα Ser 485) contributes ADR-induced VSMC senescence, not by expression of p-AMPK α Thr172.

Because the regulation of cellular senescence differs across tissues and cells, it is difficult to explain how SIRT1 and AMPK signaling might be linked to the coordinated molecular network governing vascular inflammation. Therefore, we consider it important that the mechanisms of their interactions and the consequences of their cross-regulations under senescence reducing conditions in VSMC be further investigated. Our results suggest the activity of SIRT1 and the phosphorylation of AMPK (Ser485) by PD might reduce VSMC senescence by inhibiting p-NF-κB.

In conclusion, we propose that PD might has an anti-aging effect in senescent VSMC by reducing p-NF-κB levels, and anti-aging effect of PD involves the activation of SIRT1 and AMPK. Our findings suggest the SIRT1/p-AMPK (Ser485) signaling pathway may be a critical control point in the amelioration of inflammation by PD, which has implications for the developments of novel therapeutic approaches.
